# Forecasting delay times in post-exposure prophylaxis to human animal bite injuries in Central Iran: A decision tree analysis

**DOI:** 10.14202/vetworld.2019.965-971

**Published:** 2019-07-05

**Authors:** Amir Hamta, Abedin Saghafipour, Seyed Abbas Hosseinalipour, Fatemeh Rezaei

**Affiliations:** 1 Department of Social Medicine, Faculty of Medical Sciences, Qom University of Medical Sciences, Qom, Iran; 2 Department of Public Health, Faculty of Health, Qom University of Medical Sciences, Qom, Iran; 3 Qom Provincial Health Center, Qom University of Medical Sciences, Qom, Iran; 4 Department of Social Medicine, Faculty of Medical Sciences, Jahrom University of Medical Sciences, Jahrom, Iran

**Keywords:** decision tree analysis, human animal bite injuries, Iran, post-exposure prophylaxis

## Abstract

**Background and Aim:**

Data mining in medical sciences provides countless opportunities for demonstrating hidden patterns of a data set. These patterns can help general physicians and health workers in preventing diseases. This study aimed to forecast delay times in post-exposure prophylaxis (PEP) to human animal bite injuries in central Iran using a decision tree analysis.

**Materials and Methods:**

The data of 2072 human animal bite cases were collected from Centers for Disease Control and Prevention unit of Qom Provincial Health Center, Iran from January 2017 to December 2018. The information related to animal bite incidents, including the biting animal characteristics and data on the bitten humans, was obtained by investigating the epidemiological survey forms of human animal bites. The decision tree model was applied to forecast the delay time of receiving PEP.

**Results:**

A delay of more than 48 h in the initiation of PEP was estimated among 12.73% of animal bite victims. The most important variables to predict delay time of receiving PEP were the species of biting animal, time and cause of animal bite occurrences in 24 h a day, respectively. Hence, the model showed a delay in the initiation of PEP if the biting animal was a cattle or, a carnivore, and the time of being bitten was from 7 am to 1 pm, or if the animal was carnivore and the time of being bitten was between 1 and 7 pm, and the cause of animal bite was playing with the animal.

**Conclusion:**

Based on the findings of the study on different variables affecting the initiation of PEP, the concepts related to animal bite and rabies, including the timely injection of anti-rabies vaccine to prevent rabies, it is a must to educate and train, all the people, especially housewives and students.

## Introduction

In medical and health-care surveillance, the biting of warm-blooded, domestic, or zoonotic animals (class Mammalia) that leads to scratches, puncture wounds, and crush injuries are considered as an animal bite [1]. Being bitten by animals is an important threat to human health because some subsequent infections of such a bite can be fatal (e.g., rabies); that is why the highest rate of mortality rate belongs to rabies as an infectious disease [2,3]. Rabies is a vaccine-preventable viral disease, and it can be transmitted among humans and all species of warm-blooded animals. The causative agent of this disease is well-adapted to the nervous system virus (Mononegavirales: Rhabdoviridae) that belongs to the genus *Lyssaviruses* [4]. It can be transmitted to humans through the saliva of infected hosts. Although rabies is usually a result of an animal bite, other transmission routes, such as mucous membranes, placenta, contaminated medical equipment, and organ transplantations, have been also reported [5]. The diagnosis of rabies is possible through clinical and laboratory tests [6].

Although rabies is a preventable disease by effective vaccination, the disease remains a health problem in many countries around the world, especially in Asian and African countries such as Bangladesh, Pakistan, India, and Iran [7]. Tens of thousands of deaths (approximately 60,000) occur annually in humans around the world as a result of rabies [8]. Most of the victims are residents of the developing countries in Asia and Africa. Approximately 30-50% of them are children under 15 years of age [2,9,10]. In Iran, human rabies has long existed as a zoonotic disease among wildlife, and domestic animal contamination with the rabies virus repeatedly happens [11]. Furthermore, in different parts of the world every year, more than 10 million people are being treated for rabies to prevent rabies [12]. Up to 10 million human animal bite cases from different areas in the world receive post-exposure prophylaxis (PEP) to prevent rabies annually [13].

According to the Qom Health Center’s report, one human rabies death was documented in Qom Province in 2017. In some cases, the lack of an advanced surveillance system of rabies and the delay in the initiation of PEP in animal bite injuries have led to an increase in rabies incidence [14]. Thus, if we can use the existing data of animal bites in human to predict humans’ future behaviors, such as their delay time to receive PEP, it helps to control and reduce rabies disease [15]. One of the issues that help us predict the future behaviors of people who are bitten by animals is data mining with the decision tree model. In recent years, data mining methods with the decision tree model have been used to predict medical data such as cancer, anemia, and Crimean-Congo Fever [16-18].

This study aimed to forecast delay times in PEP to human animal bite injuries in central Iran using the decision tree analysis.

## Materials and Methods

### Ethical approval

Ethical clearance was earned from the Institutional Ethics Committee of Qom University of Medical Sciences (QUMS.REC.1396.116), Iran.

### Study area and data collection

**Figure-1:**
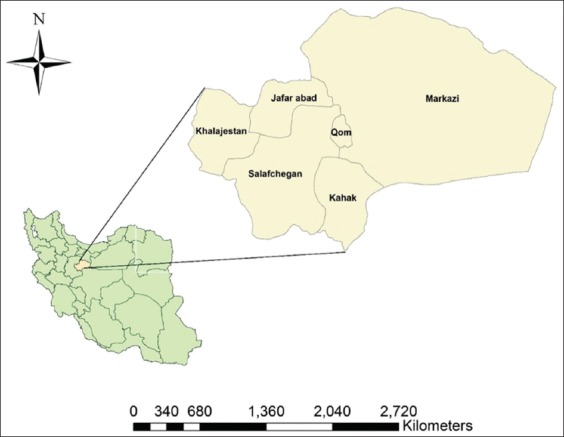
Position of Qom Province in Iran (left) and its geographical situation (right) [19].

As shown in Figure-1 [19], Qom Province is located in the arid and semi-arid region in central Iran. The data of 2072 human animal bite cases were collected from Centers for Disease Control and Prevention (CDC) unit of Qom Provincial Health Center, Iran, from January 2017 to December 2018. The information related to animal bite victims, including the biting animal characteristics and data on the bitten humans, such as age, gender, residency place, animal bite occurrence location, date of bites, and the history of reception post-bite vaccination, was obtained by investigating the epidemiological survey forms of human animal bites.

### Study design

In this study, data mining with the decision tree model was applied to detect the delay time of receiving PEP to prevent clinical rabies. The PEP, as a preventive measure of rabies, is considered a local treatment of the wounds (washing the wound immediately with soap and water, and rabies vaccination after animal bite) [20]. This preventive method is very useful when there are lots of covariates, and the sample size is large [21]. Since this is a non-parametric method of statistical analysis, some preconditions of ordinary statistical analysis such as the normality and equality of variance or sensitivity to the outliers, are not relevant, hence not needed [22]. The method was initially introduced by Quinlan [23] and has been widely used in medical science [24]. The decision tree model generates and classifies simple, interpretable results as a set of if-then rules. The structure of this classifier is exactly like a Flowchart. The root node in the tree, which is the most important predictor, is placed at the topmost decision node. Making a decision based on a decision tree is as follows: First, an attribute is selected, and logical test is done; second, each outcome of test is branched to determine the corresponding child node of each attribute; third, this procedure is recursively run; and finally, based on the termination rule, which is preventing of over fitting, partitioning would be stopped [24].

### Statistical analysis

In order to conduct decision tree analysis, determine important variables, and find the cutoff point for continuous variables, the classification and regression trees (CART) algorithm and Gini index were applied.

## Results

**Figure-2:**
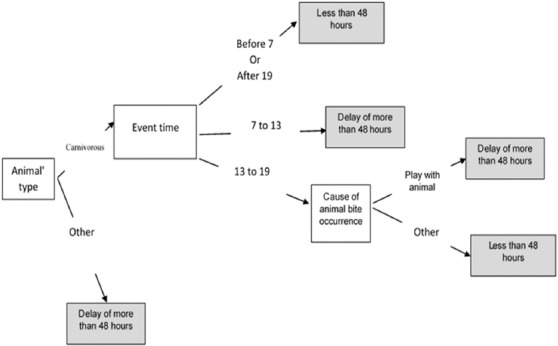
Final decision tree model for predicting delay of more than 48 h in the initiation of post-exposure prophylaxis using classification and regression trees algorithm.

Over the course of January 2017 to December 2018, 2072 cases referred to CDC unit of Qom Provincial Health Center. In this period, 211 suspected cases of the animal bites were submitted to Pasteur Institute of Iran (IPI) for rabies detection, and the disease of 8 cases was confirmed. Men were more exposed to animal bite than women (85.20% vs. 14.8%). Almost 30% of them were self-employed, students or had other jobs in the next rank. They were mostly 20-30 years old (25%). The results revealed that the prevalence of a delay by more than 48 h in the initiation of PEP was 12.73%, and after being bitten, the delayed PEP was statistically more observed in women than men (19% vs. 11.3%), and Kappa coefficient was calculated as 0.486. There was a significant relationship between the job of the cases, and their PEP as the results of Chi-square showed that the number of delayed housewives and students was significantly more than the expected values whereas drivers and businessmen were less likely to delay. The results of univariate analysis are shown in Table-1. First, for data mining based on univariate analysis, all variables that had a significant effect on the delayed PEP or were emphasized as important in the literature, were put in the model. According to the first step, sex, age, occupation, nationality, type of animal, animal status, time of event, place, number of injuries on the human body, being domestic or the stray of animal, the depth and area of injuries on human body, and the cause of animal bite occurrence were selected to be considered in the decision tree model. Next, using CART algorithm, the depth of the tree was determined to be equal to three. The Gini index, as an impurity function of CART algorithm, showed that the most important variables for predicting the delay of PEP were animal’s type, the time of getting bitten (event time), and cause of animal bite, respectively (Figure-2).

**Table 1:** Univariate analysis affecting factors on the delay of more than 48 h in the initiation of PEP.

Factors for delay	Total	Delay		p-value

		No, n(%)	Yes, n(%)
Sex				
Male	2009	1781 (88.7)	228 (11.3)	<0.001
Female	405	328 (81.0)	77 (19.0)
Occupation				
Self-employed	722	649 (89.9)	73 (10.1)	<0.001
Children	150	128 (85.3)	22 (14.7)	
Pupil	376	320 (85.1)	56 (14.9)	
housewives	236	196 (83.1)	40 (16.9)	
Rancher	149	129 (86.6)	20 (13.4)	
Student	63	47 (74.6)	16 (25.4)	
Driver	64	62 (96.9)	2 (3.1)	
Former	99	88 (88.9)	11 (11.1)	
Worker	171	148 (86.5)	23 (13.5)	
Employee	120	110 (91.7)	10 (8.3)	
Other	259	231 (89.2)	28 (10.8)
Nationality				
Iranian	2234	1958 (87.6)	276 (12.4)	0.09
Other	180	151 (83.9)	29 (16.1)
Residency place				
Rural	474	398 (84.0)	76 (16.0)	0.006
Urban	1897	1678 (88.5)	219 (11.5)
Event description				
Exposure to suspected animal rabies	19	11 (57.9)	8 (42.1)	<0.01
Animal bite	2395	2098 (87.6)	297 (12.4)
Cause of animal bite occurrence				
Teasing animals	502	440 (87.6)	62 (12.4)	<0.01
playing with the animal	344	276 (80.2)	68 (19.8)	
Human defense against animal attack	52	46 (88.5)	6 (11.5)	
Animal’s sudden attack on humans	661	598 (90.5)	63 (9.5)	
Because of feeding the animal and keeping it	370	320 (86.5)	50 (13.5)	
Because of hunting the animal	98	90 (91.8)	8 (8.2)	
Others	387	339 (87.6)	48 (12.4)
Type of animal				
Cattle(Horse, Donkey, Cow, Sheep, Camel, Goat)	75	50 (66.7)	25 (33.3)	<0.001
Carnivorous(Dog, Jackal, Pig, Fox)	1187	1060 (89.3)	127 (10.7)	
Cat	1100	968 (88.0)	132 (12.0)	
Other	52	31 (59.6)	21 (40.4)
Being stray				
No	1238	1077 (87.0)	161 (13.0)	0.32
Yes	1170	1026 (87.7)	144 (12.3)	
Status animal				
Escaped				
Yes	250	201 (80.4)	49 (19.6)	<0.01
No	842	746 (88.6)	96 (11.4)
Killed				
No	2376	2086 (87.8)	290 (12.2)	<0.01
Yes	38	23 (60.5)	15 (39.5)
Place of injury in human body				
Lower limb of the human body	693	625 (90.2)	68 (9.8)	<0.01
Upper limb of the human body	1539	1333 (86.6)	206 (13.4)	
Others	160	137 (85.6)	23 (14.4)
Number of injury in human body				
1	1311	1132 (86.3)	179 (13.7)	<0.001
2	631	557 (88.3)	74 (11.7)	
3	255	226 (88.6)	29 (11.4)	
More than 3	196	183 (93.4)	13 (6.6)
Entering the saliva of animal to human body				
Yes	2405	2103 (87.4)	302 (12.6)	0.094
No	9	6 (66.7)	3 (33.3)
Puncture wounds				
No	2131	1861 (87.3)	270 (12.7)	0.44
Yes	283	248 (87.6)	35 (12.4)
Scratches				
No	202	162 (80.2)	40 (19.8)	<0.01
Yes	2212	1947 (88.0)	265 (12.0)
Crush injuries				
No	2375	2072 (87.2)	303 (12.8)	0.11
Yes	39	37 (94.9)	2 (5.1)	
Time of event				
Before 7 am	202	188 (93.1)	14 (6.9)	<0001
7-13	661	542 (82.0)	119 (18.0)	
13-19	801	696 (86.9)	105 (13.1)	
19-24	693	643 (92.8)	50 (7.2)
Age				
<10	231	195 (84.4)	36 (15.6)	0.348
10-20	377	320 (84.9)	57 (15.1)	
20-30	612	537 (87.7)	75 (12.3)	
30-40	532	472 (88.7)	60 (11.3)	
40-50	286	254 (88.8)	32 (11.2)	
>50	376	331 (88.0)	45 (12.0)	

According to Figure-2, the following five rules can be deduced:

1. If “The animal is NOT carnivorous” then the delay time is more than 48 h.
2. If “The animal is carnivorous” and “Event time is before 7 or after 19” then the delay time is <48 h.
3. If “The animal is carnivorous” and “Event time is 7-13” then the delay time is more than 48 h.
4. If “The animal is carnivorous” and “Event time is 13-19” and “Playing with an animal” then the delay time is more than 48 h.
5. If “The animal is carnivorous” and “Event time is 13-19” and “Cause of animal bite occurrence is not playing with an animal” then the delay time is <48 h.

According to the incubation period of rabies disease, in three steps including 1, 3, and 4 that the delay time for getting PEP was more than 48 h, the risk of rabies is higher than other steps.

The performance evaluation of the proposed model for predicting the delay of more than 48 h in the initiation of PEP is shown in Table-2.

**Table 2:** Performance evaluation of the decision tree model for predicting delay of more than 48 h in the initiation of PEP.

Statistic	Value(%)	95% CI
Sensitivity	63.61	57.9369.01
Specificity	63.02	60.91-65.08
Positive predicted value	19.92	18.35-21.59
Negative predicted value	92.29	91.14-93.31
Accuracy	63.09	61.13-65.02

PEP=Post-exposure prophylaxis

## Discussion

In the present study, we estimated a delay of more than 48 h in the initiation of PEP among 12. 73% of animal bite victims. Although the incubation period of rabies in humans typically ranges from 15 days to 3 months, an average of 1-2 months (in 75% of human rabies cases <3 months) from a few days to more than 3 months have also been reported [25]. According to the national guidelines for rabies control (I.R. Iran, 2004), developed by the IPI (WHO collaborating center on rabies) and the Center for Disease Management (Zoonosis Control Department), it is vital to initiate PEP immediately, including the injection of anti-rabies vaccine and removing the rabies virus from scratches, puncture wounds, or crush injuries by washing [20]. In humans, the rabies virus can enter the peripheral nerves from scratches, puncture wounds, or crush injuries of bitten victim and then go to their central nervous system (CNS). Then, when it reaches the CNS of the bitten victim, it is deployed there and, the victim becomes infected with rabies disease. In this stage, no therapeutic measures can be effective, and the infected person will die [3]. Hence, any medical treatment that is needed to save the life of humans bitten by an animal should be done before the onset of clinical symptoms of rabies [2]. Therefore, we can conclude that approximately 13% of bite victims in the present study who had a delay in the initiation of PEP were exposed to the rabies disease. The findings of this study showed that men had more delay than women in receiving the timely anti-rabies vaccine in case of animal bites. In other previous medical studies, it has also been proved that women are more concerned with medical care than men, and they often take necessary actions to receive required health care [26]. In this study, it was also observed that men were more delayed. This study also identified that there was a significant relationship between job and delay for receiving the anti-rabies vaccine. Based on the Chi-square test, students and housewives were delayed more than the expected time. In contrast, drivers and businessmen were delayed less than expected. One of the possible reasons behind this condition may be that housewives and students are more likely to be busy than other people with different jobs, or that this group has not received sufficient training to be warned against the possible and subsequent problems of being bitten by animals [27]. Based on the decision tree analysis, it can be observed that the most important predictor variables to predict the delay time for receiving anti-rabies vaccine were the species of biting animal, time, and the cause of animal bite, respectively. The model indicates that if the species of biting animal is a cattle or, a carnivore and the time of the animal bite occurrence is from 7 am to 1 pm, or if the species of animal is a carnivore and the time of the animal bite is occurrence between 1 and 7 pm and the cause of animal bite is playing with the animal, there will be a delay in the initiation of PEP. Regarding the epidemiological condition of rabies in Iran, in all cases of an animal bite, the biting animal should be considered as a rabid animal, and the necessary medical treatment (PEP) should be conducted immediately, even if the biting animal looks calm and healthy. Because of the reasons mentioned above, PEP centers in Iran are all on the alert, 24 hours a day, and on holidays, and they provide treatment services for rabies prevention free of charge. Thus, the bitten people can go to the PEP centers at any time of the day and receive medical treatment as soon as possible [28,29]. The decision tree model in this study showed that when the biting animals are cattle, the bitten people will come to in the initiation of PEP with delay. Because many bitten people think that only carnivorous animals, like dogs, cats, and wolves, can transmit rabies, they believe that being bitten by domestic animals cannot transmit rabies, hence not dangerous for humans [20]. This presumption is wrong as it has already been proved that all mammalian warm-blooded animals, can be a reservoir and host of rabies. Furthermore, if a human being is bitten by stray animals for any reasons (animal attacks, feeding animals, or playing with animals), They need to receive PEP immediately [30]. Unfortunately, as the model suggests, some bitten people think that if animals are biting when they are playing with them, they do not need to receive anti-rabies treatment, or they come to receive an anti-rabies vaccine with delay.

## Conclusion

According to the variables of the study affecting the initiation of PEP, it is essential to educate and to train all the people, especially housewives and young students, so that they can immediately refer to the health centers to receive a timely injection of anti-rabies vaccine to prevent possible rabies. It is recommended that further research is carried out on the reduction of human rabies with the existing data that have been collected at PEP centers in other provinces of the country. By so doing, the results will be made available to the Ministry of Health to have a better plan and formulate more comprehensive guidelines for rabies control.

## Authors’ Contributions

AH designed the study. AS and SAH gathered the data. AH and FR interpreted the results and analyzed the data. AS drafted and revised the manuscript. All authors read and approved the final manuscript.

## References

[1] Rothen K, Tsokos M, Handrick W (2015). Animal and human bite wounds. Dtsch. Arztebl. Int.

[2] World Health Organization.

[3] Mahadevan A, Suja M.S, Mani R.S, Shankar S.K (2016). Perspectives in diagnosis and treatment of rabies viral encephalitis:Insights from pathogenesis. Neurotherapeutics.

[4] Rupprecht C, Kuzmin I, Meslin F (2017). Lyssaviruses and rabies:Current conundrums, concerns, contradictions and controversies. F1000 Research.

[5] Zhu J, Pan J, Lu Y.A (2015). Case report on indirect transmission of human rabies. J. Zhejiang Univ. Sci. B.

[6] Mani R.S, Madhusudana S.N (2013). Laboratory diagnosis of human rabies:Recent advances. Sci. World J 2013.

[7] Abraham S, Ravindran J, Abishaw N, Sandam N.P, Thimmareddy P, Govindaraju G (2017). Review on rabies and vaccines. Int. J. Curr. Microbiol. Appl. Sci.

[8] World Health Organization (2018). What is Rabies?.

[9] World Health Organization (WHO) (2013). WHO Expert Consultation on Rabies.

[10] Rabies http://www.who.int/news-room/fact-sheets/detail/rabies.

[11] Janani A.R, Fayaz A, Simani S, Farahtaj F, Eslami N, Howaizi N, Biglari P, Sabetghadam M (2008). Epidemiology and control of rabies in Iran. Dev. Biol. (Basel).

[12] World Health Organization (2010). Rabies vaccines: WHO position paper. Wkly. Epidemiol. Rec.

[13] Ngugi J.N, Maza A.K, Omolo O.J, Obonyo M (2018). Epidemiology and surveillance of human animal-bite injuries and rabies post-exposure prophylaxis, in selected counties in Kenya 2011-2016. BMC Public Health.

[14] Hampson K, Dobson A, Kaare M, Dushoff J, Magoto M, Sindoya E (2008). Rabies exposures, post-exposure prophylaxis and deaths in a region of endemic canine rabies. PLoS Negl. Trop. Dis.

[15] Esmaeilzadeh F, Rajabi A, Vahedi S, Shamsadiny M, Ghojogh M.G, Hatam N (2017). Epidemiology of animal bites and factors associated with delays in initiating post-exposure prophylaxis for rabies prevention among animal bite cases:A population-based study. J. Prev. Med. Public Health.

[16] Nejad A.S, Saeid A.H, Rose I.M, Rowhanimanesh A.R (2014). Modeling a data mining decision tree and propose a new model for the diagnosis of skin cancer by immunohistochemical staining methods. J. Health Biomed. Inform.

[17] Safaee P, Noorossana R, Heidari K, Soleimani P (2016). Using decision tree to predict serum ferritin level in women with anemia. Tehran Univ. Med. J.

[18] Gohari R.E, Gohari E.E, Shafiei M (2017). Detection of Crimean-congo fever using C4.5. decision tree. J. Health Biomed. Inform.

[19] Saghafipour A, Vatandoost H, Zahraei-Ramazani A.R, Yaghoobi-Ershadi M.R, Rassi Y, Shirzadi M.R, Akhavan A.A (2017). Spatial distribution of phlebotomine sand fly species (Diptera:Psychodidae) in Qom Province, Central Iran. J. Med. Entomol.

[20] Sharifian J, Simani S, Shirzadi M.R, Faiias A, Hooshmand B, National Guideline for Control and Prevention of Rabies (2004).

[21] Hosseini M, Tazhibi M, Amini M, Zareei A, Jahani-Hashemi H (2010). Using classification tree for prediction of diabetic retinopathy on Type II diabetes. J. Isfahan Med. Sch.

[22] Sheskin D.J (2000). Parametric and Nonparametric Statistical Procedures.

[23] Quinlan J.R (1986). Induction of decision trees. Mach. Learn.

[24] Podgorelec V, Kokol P, Stiglic B, Rozman I (2002). Decision trees:An overview and their use in medicine. J. Med. Syst.

[25] Salomão C, Nacima A, Cuamba L, Gujral L, Amiel O, Baltazar C, Cliff J, Gudo E.S (2017). Epidemiology, clinical features and risk factors for human rabies and animal bites during an outbreak of rabies in Maputo and Matola cities, Mozambique 2014:Implications for public health interventions for rabies control. PLoS Negl. Trop. Dis.

[26] Thompson A.E, Anisimowicz Y, Miedema B, Hogg W, Wodchis W.P, Aubrey-Bassler K (2016). The influence of gender and other patient characteristics on health care-seeking behavior:A QUALICOPC study. BMC Fam. Pract.

[27] Riabi H.R.A, Ghorbannia R, Mazlum S.B, Atarodi A (2015). A three-year (2011–2013) surveillance on animal bites and victims vaccination in the South of Khorasan-e-Razavi Province, Iran. J. Clin. Diagn. Res.

[28] Khazaei S, Rezaeian S, Soheylizad M, Gholamaliee B (2014). Factors associated with delay in post-exposure prophylaxis in bitten people. Med. J. Islam. Repub. Iran.

[29] Han M.G, Sang R.J, Jeong Y.E, Ju Y.R, Cho J.E, Park J.S (2012). Epidemiologic features of animal bite cases occurring in rabies-endemic areas of Korea 2005 to 2009. Osong Public Health Res. Perspect.

[30] Kabeta T, Deresa B, Tigre W, Ward M.P, Mor S.M (2015). Knowledge, attitudes and practices of animal bite victims attending an anti-rabies health center in Jimma town, Ethiopia. PLoS Negl. Trop. Dis.

